# Evaluating Blood Culture Parameters to Identify Patients at Low Risk of Infective Endocarditis Among Those With Bacteremia by Gram-positive Cocci

**DOI:** 10.1093/ofid/ofaf518

**Published:** 2025-08-22

**Authors:** Nicolas Fourré, Virgile Zimmermann, Nicoleta Ianculescu, Thomas Brahier, Zélie Dennebouy, André Teixeira-Antunes, Pierre Monney, Georgios Tzimas, Laurence Senn, Lars Niclauss, Matthias Kirsch, Benoit Guery, Matthaios Papadimitriou-Olivgeris

**Affiliations:** Infectious Diseases Service, Lausanne University Hospital and University of Lausanne, Lausanne, Switzerland; Infectious Diseases Service, Lausanne University Hospital and University of Lausanne, Lausanne, Switzerland; Department of Cardiology, Lausanne University Hospital and University of Lausanne, Lausanne, Switzerland; Infectious Diseases Service, Lausanne University Hospital and University of Lausanne, Lausanne, Switzerland; Infectious Diseases Service, Lausanne University Hospital and University of Lausanne, Lausanne, Switzerland; Infectious Diseases Service, Lausanne University Hospital and University of Lausanne, Lausanne, Switzerland; Department of Cardiology, Lausanne University Hospital and University of Lausanne, Lausanne, Switzerland; Department of Cardiology, Lausanne University Hospital and University of Lausanne, Lausanne, Switzerland; Infectious Diseases Service, Lausanne University Hospital and University of Lausanne, Lausanne, Switzerland; Infection Prevention and Control Unit, Lausanne University Hospital and University of Lausanne, Lausanne, Switzerland; Department of Cardiac Surgery, Lausanne University Hospital and University of Lausanne, Lausanne, Switzerland; Department of Cardiac Surgery, Lausanne University Hospital and University of Lausanne, Lausanne, Switzerland; Infectious Diseases Service, Lausanne University Hospital and University of Lausanne, Lausanne, Switzerland; Infectious Diseases Service, Lausanne University Hospital and University of Lausanne, Lausanne, Switzerland; Infectious Diseases Service, Hospital of Valais and Institut Central des Hôpitaux, Sion, Switzerland

**Keywords:** bacteremia, *Enterococcus faecalis*, infective endocarditis, *Staphylococcus aureus*, streptococci

## Abstract

**Background:**

Identifying patients at low risk for infective endocarditis (IE) among those with bacteremia by Gram-positive cocci is critical to optimize cardiac imaging use. The aim was to assess the diagnostic performance of blood culture parameters in identifying patients at low risk for IE.

**Methods:**

Adult patients with bacteremia due to *Staphylococcus aureus*, streptococci, or *Enterococcus faecalis* at the Lausanne University Hospital were included. Low-risk criteria were defined as: only one positive out of four initial blood culture bottles and bacteremia clearance within 48 hours. The primary outcome was the diagnosis of IE, determined by the Endocarditis Team. Negative likelihood ratios (NLRs) were calculated.

**Results:**

Among 2165 episodes of bacteremia, 1165 (54%) were due to *S. aureus*, 726 (34%) to streptococci, and 326 (15%) to *E. faecalis*. IE was diagnosed in 561 (26%) episodes. Among all episodes, 1767 (82%) had >1 positive out of the initial 4 blood culture bottles collected, and 1783 (82%) had either >1 positive out of the initial 4 blood culture bottles or persistent bacteremia for ≥48 hours. Having only 1 positive out of 4 initial blood culture bottles was associated with a NLR of 0.10 (95% CI, .06–.18). When combining both criteria, 1 positive out of 4 blood culture bottles and bacteremia clearance before 48 hours, the NLR was 0.08 (0.05–0.15).

**Conclusions:**

Simple blood culture parameters may help identify patients at low risk for IE. However, the approach classifies most patients as high-risk and may have limited impact on reducing echocardiography use.

## Teaser

In this retrospective cohort study of 2165 adults with bacteremia, the combination of only one positive out of four initial blood culture bottles and clearance of bacteremia within 48 hours was associated with a low negative likelihood ratio for IE across all pathogen groups. However, 83% of episodes were classified as high-risk.

*Staphylococcus aureus*, streptococci, and *Enterococcus faecalis* are among the most common causes of infective endocarditis (IE) and are classified as typical IE pathogens in the Duke criteria [1–3]. Diagnosing or excluding IE in patients with bacteremia due to these Gram-positive cocci is often challenging and frequently requires an extensive diagnostic workup, including echocardiography, as well as, advanced imaging modalities such as ^18^F-fluorodeoxyglucose positron emission tomography/computed tomography (^18^F-FDG PET/CT) or cardiac CT [1].

Given the risk of IE, the 2023 European Society of Cardiology (ESC) guidelines recommend routine echocardiographic evaluation for all patients with bacteremia caused by *S. aureus*, *E. faecalis*, and certain streptococcal species [1]. However, the utility of universal echocardiographic screening in this context remains uncertain. To better stratify risk and reduce unnecessary cardiac imaging, several scoring systems have been proposed: VIRSTA, POSITIVE, PREDICT, LAUSTAPHEN, and SABIER for *S. aureus* bacteremia [4–8], HANDOC for streptococci [9, 10], and NOVA and DENOVA for enterococcal bacteremia [11, 12]. Despite their intent, most of these scores have significant limitations. Some are difficult to implement in clinical practice due to the number of required variables (VIRSTA, SABIER), complex point allocations (VIRSTA, POSITIVE, PREDICT, SABIER, NOVA) or performance issues such as overestimating the risk of IE (VIRSTA, NOVA), or misclassifying many IE cases as low risk (POSITIVE, PREDICT) [4–6, 8, 11]. These shortcomings hinder their routine clinical use.

The association of IE with several blood culture-derived variables, including persistent bacteremia or presence of multiple positive blood culture sets, has been previously demonstrated [9–14]. In contrast, the number of positive blood culture bottles has rarely been linked to IE risk; however, low-grade *S. aureus* bacteremia, defined as a single positive blood culture bottle, has been associated with a reduced risk of IE [15]. Another blood culture-derived variable that has been explored is the time to blood culture positivity (TTP), which serves as an indicator of the microbial load of the infecting microorganism. This association has been observed primarily in *S. aureus* bacteremia [6, 7], whereas studies on streptococcal infections have found no such association [16], and findings in *E. faecalis* bacteremia have been inconsistent [17, 18]. A novel, simplified scoring approach was recently proposed, based solely on two blood culture-derived variables: (1) the number of positive bottles in the initial blood culture set, and (2) persistence of bacteremia for 2 days [19]. This method offers the advantage of applicability across different Gram-positive cocci, including *S. aureus*, streptococci, and *E. faecalis*. Notably, the combination of only 1 positive bottle out of four and clearance of bacteremia within 48 hours was associated with a low negative likelihood ratio (NLR) for IE across all pathogen groups [19].

The aim of this study is to evaluate the performance of the number of positive blood culture bottles and persistence of bacteremia in identifying patients with bacteremia due to *S. aureus*, streptococci or *E. faecalis* who are at low risk for IE.

## METHODS

This retrospective study was conducted at Lausanne University Hospital (CHUV), Switzerland, between January 2015 and June 2024, and comprised 2 distinct cohorts: a retrospective bacteremia cohort (January 2015–December 2021) and a prospective cohort of patients with suspected IE (January 2022–June 2024). The study was approved by the ethics committee of the Canton of Vaud (CER-VD 2021-02516, CER-VD 2017-02137). Suspected IE was defined as the combination of blood cultures being drawn and echocardiography being performed specifically to investigate IE.

Inclusion criteria were adult patients (≥18 years old), at least one blood culture positive for *S. aureus*, streptococci, or *E. faecalis* and no documented refusal for use of clinical data. Exclusion criteria consisted of patients with only one set of blood cultures at initial collection, antibiotic treatment prior to initial blood culture collection, polymicrobial bacteremia, lack of follow-up blood cultures until clearance, and incomplete medical records—such as patients transferred to another hospital, transitioned to limitation of care, or who died before sufficient diagnostic evaluation to assess for IE.

Data on demographics, clinical presentation, imaging, microbiology (including number of positive blood culture bottles from initial cultures and persistence of bacteremia), surgery, and pathology were manually extracted from electronic health records by internal medicine and infectious diseases (ID) consultants. All data were reviewed by an ID consultant (M. P. O.).

At CHUV, ID consultation was systematically performed for patients with *S. aureus* bacteremia or suspected IE, regardless of the pathogen [20]. For streptococcal or enterococcal bacteremia without suspicion of IE, ID consultation was at the discretion of the attending physician [9, 21]. Follow-up blood cultures were also systematically performed for patients with *S. aureus* bacteremia or clinical suspicion of IE [9, 20, 21]. Cases were diagnosed as IE or no-IE by the Endocarditis Team (from January 2018 to June 2024) or by expert clinicians (2015–2017; M. P. O., P. M.); this diagnosis was used as the reference standard. IE cases were further classified as definite or possible IE based on the 2023 International Society of Cardiovascular Infectious Diseases (ISCVID) Duke criteria [2]. An infection site other than IE was determined by an ID consultant based on a comprehensive assessment of clinical, radiological, microbiological, and surgical findings.

The date of the first positive blood culture was considered the onset of infection. A new episode was recorded if more than 30 days had passed since the completion of antimicrobial therapy for a prior episode. TTP was defined as the time (hours), from the start of incubation to the detection of microbial growth in the first positive blood culture bottle during a given bacteremic episode. When multiple bottles were collected, the fastest TTP was used for analysis. Persistent bacteremia was defined as positive blood cultures for at least 48 hours following the initial collection. Streptococci were classified into low- and high-risk categories. Low-risk streptococcal species included *Streptococcus pneumoniae*, *Streptococcus pyogenes*, *Streptococcus agalactiae*, *Streptococcus dysgalactiae*, *Streptococcus salivarius*, *Streptococcus anginosus*, *Streptococcus constellatus*, and *Streptococcus thermophilus*. All other streptococcal species were considered high risk.

Statistical analyses were performed using SPSS version 26.0 (SPSS Inc., Chicago, IL, USA). Categorical variables were compared using the χ^2^ test or Fisher's exact test, while continuous variables were analyzed using the Mann–Whitney U test. Three variables were evaluated for their ability to identify episodes at high risk for IE, by assessing agreement with the reference diagnosis (Endocarditis Team or expert clinician assessment): (1) > 1 positive out of 4 initial blood culture bottles, (2) persistent bacteremia for at least 48 hours, and (3) either >1 positive out of 4 initial blood culture bottles or persistent bacteremia for at least 48 hours. For each, sensitivity, specificity, positive, and negative predictive values (PPV, NPV), positive likelihood ratio, and NLR were calculated with 95% confidence intervals (CI). All tests were two-tailed, and a *P*-value of <.05 was considered statistically significant.

## RESULTS

**Figure 1.**
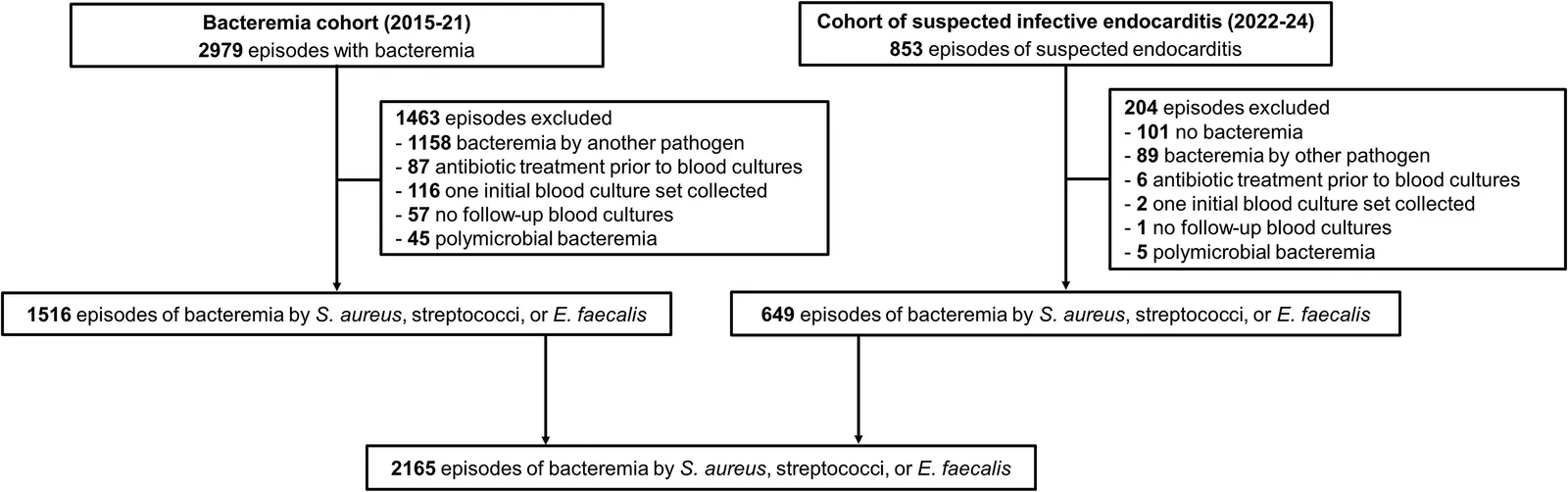
Flowchart of included episodes.

Among 3832 episodes of bacteremia from both cohorts, 2165 episodes were included (Figure 1), of which 1165 (54%) were due to *S. aureus* (82; 7% being methicillin-resistant), 274 (13%) to low-risk streptococci, 400 (19%) to high-risk streptococci, and 326 (15%) to *E. faecalis*.

Cardiac imaging was performed in 1736 (80%) episodes; transthoracic echocardiography in 1609 (74%), transesophageal echocardiography (TEE) in 905 (42%), ^18^F-FDG PET/CT in 279 (13%), and cardiac CT in 45 (2%) episodes. ID consultation was provided in 2002 (93%) episodes.

IE was diagnosed by the Endocarditis Team or expert clinicians in 561 (26%) episodes. Of these, 392 (70%) involved native valves, 136 (24%) prosthetic valves, and 60 (11%) cardiac implantable electronic device (CIED) leads. According to the 2023 ISCVID Duke criteria, 450 (80%) of these IE cases were classified as definite IE, and the remaining 111 (20%) as possible IE.

Among all 2165 episodes, 1767 (82%) had >1 positive out of the initial 4 blood culture bottles collected: 980 (84%) of 1165 *S. aureus*, 233 (85%) of 274 low-risk streptococci, 287 (72%) of 400 high-risk streptococci, and 267 (82%) of 326 *E. faecalis* (Table 1). Persistent bacteremia for at least 48 hours was observed in 407 episodes (19%); 359 (31%) with *S. aureus*, 4 (2%) with low-risk streptococci, 8 (2%) with high-risk streptococci, and 35 (11%) with *E. faecalis*. Based on the presence of either >1 positive out of the initial 4 blood culture bottles or persistent bacteremia for ≥48 hours, 1783 (82%) episodes were classified as high-risk for IE; 990 (85%) with *S. aureus*, 235 (86%) with low-risk streptococci, 287 (72%) with high-risk streptococci, and 270 (83%) with *E. faecalis*. In episodes of *S. aureus* bacteremia, TTP was significantly shorter in patients with IE compared to those without (median 10 hours vs 13 hours; *P* < .001). No significant difference in TTP was observed for low- or high-risk streptococci or for *E. faecalis*.

**Table 1.** Comparison of Episodes With or Without Infective Endocarditis Among 2165 Episodes With Bacteremia by Either *S. Aureus*, Streptococci or *E. Faecalis*

	No Infective Endocarditis (n = 1604)	Infective Endocarditis (n = 561)	*P*
Male sex, n (%)	1111 (69)	416 (74)	.031
Age (y), median (IQR)	68 (57–78)	69 (55–78)	.870
Cardiac predisposition			
Intravenous drug use, n (%)	78 (5)	54 (10)	<.001
Prior infective endocarditis, n (%)	26 (2)	53 (9)	<.001
Prosthetic valve; surgical or transcatheter replacement, n (%)	76 (5)	159 (28)	<.001
Cardiac implantable electronic devices, n (%)	116 (7)	97 (17)	<.001
Manifestations			
Fever, n (%)	1360 (85)	476 (85)	1.000
Embolic events, n (%)	86 (5)	325 (58)	<.001
Immunological phenomena, n (%)	11 (0.7)	42 (8)	<.001
Hematogenous native bone and joint infections, n (%)	183 (11)	114 (20)	<.001
*S. aureus*, n (%)	881 (55)	284 (51)	.085
Time to positivity (h),^a^ median (IQR)	13 (10–17)	10 (8–13)	<.001
Number of positive bottles out of initial blood culture bottles	…	…	<.001
1, n (%)	177 (20)	8 (3)	
2 from same blood culture set, n (%)	68 (8)	6 (2)	
2 from different blood culture set, n (%)	105 (12)	24 (8)	
3, n (%)	92 (10)	13 (5)	
4, n (%)	439 (50)	233 (82)	
Persistent bacteremia ≥48 h, n (%)	195 (22)	164 (58)	<.001
>1 positive bottle or persistent bacteremia ≥48 h, n (%)	712 (81)	278 (98)	<.001
Low-risk streptococci,^b^ n (%)	229 (14)	45 (8)	<.001
Time to positivity (h),^c^ median (IQR)	11 (8–17)	10 (7–14)	.154
Number of positive bottles out of initial blood culture bottles	…	…	<.001
1, n (%)	40 (18)	1 (2)	
2 from same blood culture set, n (%)	26 (11)	0 (0)	
2 from different blood culture set, n (%)	33 (14)	7 (16)	
3, n (%)	26 (11)	2 (4)	
4, n (%)	104 (46)	35 (78)	
Persistent bacteremia ≥48 h, n (%)	4 (2)	0 (0)	1.000
>1 positive bottle or persistent bacteremia ≥48 h, n (%)	191 (83)	44 (98)	.009
High-risk streptococci,^d^ n (%)	265 (17)	135 (24)	<.001
Time to positivity (h),^e^ median (IQR)	12 (10–16)	12 (10–14)	.260
Number of positive bottles out of initial blood culture bottles	…	…	<.001
1, n (%)	110 (42)	3 (2)	
2 from same blood culture set, n (%)	32 (12)	3 (2)	
2 from different blood culture set, n (%)	41 (15)	14 (10)	
3, n (%)	12 (5)	5 (4)	
4, n (%)	70 (26)	110 (82)	
Persistent bacteremia ≥48 h, n (%)	3 (1)	5 (4)	.126
>1 positive bottle or persistent bacteremia ≥48 h, n (%)	155 (59)	132 (98)	<.001
*E. faecalis*, n (%)	229 (14)	97 (17)	.087
Time to positivity (h),^f^ median (IQR)	12 (9–14)	11 (9–13)	.137
Number of positive bottles out of initial blood culture bottles	…	…	<.001
1, n (%)	57 (25)	2 (2)	
2 from same blood culture set, n (%)	21 (9)	5 (5)	
2 from different blood culture set, n (%)	41 (18)	1 (1)	
3, n (%)	25 (11)	4 (4)	
4, n (%)	85 (37)	85 (88)	
Persistent bacteremia ≥48 h, n (%)	25 (11)	10 (10)	1.000
>1 positive bottle or persistent bacteremia ≥48 h, n (%)	175 (76)	95 (98)	<.001

Abbreviation: IQR, interquartile range.

^a^Time to blood culture positivity for *S. aureus* bacteremia was available in 1069/1165 (92%) episodes.

^b^ *S. pneumoniae*, *S. pyogenes*, *S. agalactiae*, *S. dysgalactiae*, *S. salivarius*, *S. anginosus*, *S. constellatus*, and *S. thermophilus*.

^c^Time to blood culture positivity for low-risk streptococcal bacteremia was available in 242/274 (88%) episodes.

^d^All other streptococci not classified as low-risk.

^e^Time to blood culture positivity for high-risk streptococcal bacteremia was available in 362/400 (91%) episodes.

^f^Time to blood culture positivity for *E. faecalis* bacteremia was available in 295/326 (90%) episodes.

Table 2 presents the diagnostic performance of this classification. Among all episodes, having only 1 positive out of 4 initial blood culture bottles was associated with a NLR of 0.10 (95% CI, .06–.18); stratified by pathogen: 0.14 (0.07–0.28) for *S. aureus*, 0.13 (0.02–0.90) for low-risk streptococci, 0.05 (0.02–0.17) for high-risk streptococci, and 0.08 (0.02–0.34) for *E. faecalis*. When combining both criteria, 1 positive out of 4 blood culture bottles and bacteremia clearance before 48 hours, the NLR was 0.08 (0.05–0.15); stratified by pathogen: 0.11 (0.05–0.25) for *S. aureus*, 0.13 (0.02–0.95) for low-risk streptococci, 0.05 (0.02–0.17) for high-risk streptococci, and 0.09 (0.02–0.35) for *E. faecalis*.

**Table 2.** Performance of Number of at Least 2 Positive out of 4 Initial Blood Culture Bottles or Persistent Bacteremia ≥48 Hours for the Diagnosis of Infective Endocarditis Among 2165 Episodes With Bacteremia by Either *S. Aureus*, Streptococci or *E. Faecalis*

	Sensitivity % (95% CI)	Specificity % (95% CI)	PPV % (95% CI)	NPV % (95% CI)	PLR % (95% CI)	NLR % (95% CI)
*S. aureus*						
>1 positive bottles	97 (95–99)	20 (17–23)	28 (27–29)	96 (92–98)	1.22 (1.17–1.26)	.14 (.07–.28)
Persistent bacteremia ≥48 h	58 (52–64)	78 (75–81)	46 (42–50)	85 (83–87)	2.61 (2.23–3.06)	.54 (.47–.62)
>1 positive bottle or persistent bacteremia ≥48 h	98 (95–99)	19 (17–22)	28 (27–29)	97 (93–98)	1.21 (1.17–1.26)	.11 (.05–.25)
Low-risk streptococci^a^						
>1 positive bottles	98 (88–100)	17 (13–23)	19 (18–20)	98 (85–100)	1.18 (1.10–1.28)	.13 (.02–.90)
Persistent bacteremia ≥48 h	0 (0–8)	98 (96–100)	…	83 (83–84)	…	1.02 (1.00–1.04)
>1 positive bottle or persistent bacteremia ≥48 h	98 (88–100)	17 (12–22)	19 (18–20)	97 (84–100)	1.17 (1.09–1.26)	.13 (.02–.95)
High-risk streptococci^b^						
>1 positive bottles	98 (94–100)	42 (36–48)	46 (43–49)	97 (92–99)	1.67 (1.51–1.86)	.05 (.02–.17)
Persistent bacteremia ≥48 h	4 (1–8)	99 (97–100)	63 (29–87)	67 (66–68)	3.27 (.79–13.49)	.97 (.94–1.01)
>1 positive bottle or persistent bacteremia ≥48 h	98 (94–100)	42 (36–48)	46 (43–49)	97 (92–99)	1.67 (1.51–1.86)	.05 (.02–.17)
*E. faecalis*						
>1 positive bottles	98 (93–100)	25 (19–31)	35 (34–37)	97 (88–99)	1.30 (1.20–1.41)	.08 (.02–.34)
Persistent bacteremia ≥48 h	10 (5–18)	89 (84–93)	29 (17–44)	70 (68–72)	0.94 (0.47–1.89)	1.01 (.93–1.09)
>1 positive bottle or persistent bacteremia ≥48 h	98 (93–100)	24 (18–30)	35 (33–37)	96 (87–99)	1.28 (1.19–1.38)	.09 (.02–.35)
All pathogens						
>1 positive bottles	98 (96–99)	24 (22–26)	31 (30–32)	96 (94–98)	1.28 (1.24–1.32)	.10 (.06–.18)
Persistent bacteremia ≥48 h	32 (28–36)	86 (84–88)	44 (40–48)	72 (70–74)	2.27 (1.91–2.69)	.79 (.74–.84)
>1 positive bottle or persistent bacteremia ≥48 h	98 (97–99)	23 (21–26)	31 (30–31)	97 (95–98)	1.28 (1.24–1.31)	.08 (.05–.15)

Abbreviations: NLR, negative likelihood ratio; NPV, negative predictive value; PLR, positive likelihood ratio; PPV, positive predictive value.

^a^ *S. pneumoniae*, *S. pyogenes*, *S. agalactiae*, *S. dysgalactiae*, *S. salivarius*, *S. anginosus*, *S. constellatus*, and *S. thermophilus*.

^b^All other streptococci not classified as low-risk.

Among the 382 episodes classified as low-risk for IE based the combination of only 1 positive out of 4 blood culture bottles and bacteremia clearance before 48 hours (Table 3), 26 (7%) had a prosthetic valve, 23 (6%) had a CIED, and 17 (5%) experienced embolic events. Twelve episodes (3%) among these low-risk cases were ultimately diagnosed with IE.

**Table 3.** Comparison of Episodes Classified as Low- or High-risk for Infective Endocarditis Among 2165 Episodes With Bacteremia by Either *S. Aureus*, Streptococci or *E. faecalis*

	Low-risk^a^ (n = 382)	High-risk^a^ (n = 1783)	*P V*alue
Male sex, n (%)	261 (68)	1266 (71)	.294
Age (y), median (IQR)	66 (55–77)	69 (56–78)	.270
Cardiac predisposition			
Intravenous drug use, n (%)	17 (5)	115 (6)	.157
Prior infective endocarditis, n (%)	7 (2)	72 (4)	.035
Prosthetic valve; surgical or transcatheter replacement, n (%)	26 (7)	209 (12)	.005
Cardiac implantable electronic devices, n (%)	23 (6)	190 (11)	.004
Manifestations			
Fever, n (%)	310 (81)	1526 (86)	.034
Embolic events, n (%)	17 (5)	394 (22)	<.001
Immunological phenomena, n (%)	1 (0.3)	52 (3)	.001
Hematogenous native bone and joint infections, n (%)	17 (5)	280 (16)	<.001
Pathogens			
*S. aureus*, n (%)	175 (46)	990 (56)	.001
Low-risk streptococci,^b^ n (%)	39 (10)	235 (13)	.127
High-risk streptococci,^c^ n (%)	113 (30)	287 (16)	<.001
*E. faecalis*, n (%)	55 (14)	271 (15)	.753
Infective endocarditis, n (%)	12 (3)	549 (31)	<.001

Abbreviation: IQR, interquartile range.

^a^High-risk was considered those that had at least 2 positive out of 4 initial blood culture bottles or persistent bacteremia for at least 48 h.

^b^ *S. pneumoniae*, *S. pyogenes*, *S. agalactiae*, *S. dysgalactiae*, *S. salivarius*, *S. anginosus*, *S. constellatus*, and *S. thermophilus*.

^c^All other streptococci not classified as low-risk.

## DISCUSSION

In this combined cohort, the presence of either only 1 positive out of 4 initial blood culture bottles or clearance of bacteremia within 48 hours among patients with bacteremia due to *S. aureus*, streptococci, or *E. faecalis* was associated with a low NLR for IE, supporting its potential value in ruling out IE.

In the original study by Freling et al [19], these same characteristics showed low NLRs (≤0.12) across all evaluated pathogen groups. Our findings are broadly consistent, but there are several important differences. First, unlike Freling et al [19], who analyzed methicillin-susceptible and -resistant *S. aureus* separately, we performed a combined analysis. This was justified as methicillin-resistant *S. aureus* accounted for only 7% of our isolates. Second, while Freling et al [19] used the 2015 ESC Duke criteria to define IE, our study relied on adjudication by the Endocarditis Team or expert clinicians. Given the known limitations of the 2015 version, such as its lower sensitivity and PPV compared to the 2023 ISCVID and ESC versions of the Duke criteria [9, 20, 22–24], our approach offers a more robust diagnostic reference, given the absence of a definitive gold standard for IE diagnosis, as diagnosing IE requires a multidisciplinary and highly specialized evaluation to ensure accuracy and optimize management. Indeed, in Freling et al [19], 35% of IE cases remained in the “possible IE” category.

Another key difference lies in cohort selection. In Freling et al [19], the control group (no IE) appeared skewed toward patients at very low risk, with relatively few patients with prosthetic valves (1%) or CIEDs (2%), which may have inflated the score's diagnostic performance. In our study, these proportions were higher (5% and 7%, respectively), reflecting a broader and more representative clinical population. We included all bacteremic episodes with at least two initial blood culture sets drawn and follow-up cultures until clearance, thus reducing selection bias and allowing for more generalizable conclusions.

The principal advantage of Freling et al's [19] score is its simplicity, relying on just two blood culture-derived parameters. In contrast, previous scores such as VIRSTA, SABIER, POSITIVE, PREDICT, and NOVA include numerous variables and complex point allocations, making them less feasible in real-world settings [4–6, 8, 11]. However, a major limitation of the Freling *et al*.'s score is its classification of over 80% of patients in the high-risk group [19]. The actual figure may be even higher, given that 7% of patients classified as low-risk had prosthetic valves, 6% had CIEDs, and 5% had embolic events, features that are highly associated with IE [1, 2, 13, 14]. Thus, patients with a typical IE pathogen and either prosthetic intracardiac material or an embolic event should be considered at high risk for IE regardless of the score, and TEE remains warranted in such cases. This over-classification is a common limitation shared with the VIRSTA and NOVA scores [5, 11].

Another concern is that persistent bacteremia was rare in cases with only one positive blood culture bottle, indicating that the score's predictive ability rests primarily on the number of initially positive bottles. This was echoed in prior work showing that more than one positive set in the initial blood cultures was independently associated with persistent bacteremia [25].

Previously developed scores for predicting IE in patients with bacteremia caused by typical IE microorganisms have incorporated blood culture–derived variables [4–7, 9–12]. The use of the number of positive blood culture sets has been validated in several pathogen-specific predictive scores, such as NOVA and DENOVA for enterococci, and HANDOC for streptococci [9–12]. Persistent bacteremia is a key element of *S. aureus* predictive scores such as VIRSTA, PREDICT, POSITIVE, and LAUSTAPHEN, reflecting the pathogen's capacity to cause more complicated, deep-seated infections [4–7]. However, while the association between IE and persistent bacteremia is well established [13, 14], other factors, such as uncontrolled infection foci, inappropriate antibiotic therapy, or failure to perform indicated surgical source control, could also lead to persistently positive follow-up blood cultures [25].

Although follow-up blood cultures are routinely performed in our center as part of the management of patients with *S. aureus* bacteremia or those suspected IE [7, 26, 27], this is not standard practice in all centers, particularly for cases of streptococcal or enterococcal bacteremia. As previously reported, follow-up cultures were obtained in 98% of *S. aureus*, 75% of streptococcal, and 81% of enterococcal bacteremia cases at our institution [7, 26]. Notably, persistent bacteremia is uncommon in streptococcal bacteremia, even when IE is present, raising questions about the utility of follow-up cultures in this context [25].

In the present study, TTP was associated with IE only in cases of *S. aureus* bacteremia, with no such association observed for bacteremia by streptococci, or *E. faecalis*, echoing previous findings [6, 7, 16–18]. In addition to this limited discriminatory capacity, TTP has been shown to vary significantly among different streptococcal species [16, 26]. Furthermore, discrepancies in TTP findings across studies may be explained by differences in blood culture collection protocols and delays between sample collection and incubation, which vary between centers. These pre-analytical factors introduce variability and limit the reproducibility and interpretability of TTP across settings. Therefore, TTP, whether used alone or in combination with other blood culture-derived variables, cannot reliably predict IE across such broad pathogen categories.

This study has several limitations. First, it is a retrospective single-center study conducted in a setting with structured ID consultation and IE evaluation pathways, which may limit generalizability. However, all data manually extracted from electronic health records were reviewed by an ID specialist, ensuring data quality and clinical accuracy. Furthermore, the study is triple the size of the original study, strengthening the robustness of our findings [19]. Second, the inclusion of patients from CHUV's suspected IE cohort likely led to an overrepresentation of IE cases. Third, cardiac imaging was not performed in 20% of episodes. Nevertheless, the risk of missed IE diagnosis was likely low: ID consultations were provided in 93% of episodes, IE diagnoses were adjudicated by specialized Endocarditis Team, and the majority of patients not diagnosed with IE received short-course antibiotic therapy (≤2 weeks) with no evidence of IE during 120 days of follow-up [9, 20, 21].

The combination of only one positive out of four initial blood culture bottles and clearance of bacteremia within 48 hours appears to effectively identify bacteremic episodes due to *S. aureus*, streptococci, and *E. faecalis* with a low probability of IE. However, the classification of over 80% of patients as high-risk undermines the score's utility in reducing unnecessary echocardiograms. Additionally, the presence of patients with prosthetic intracardiac material or embolic events in the low-risk group further limits its safe implementation in clinical practice. Prospective studies are warranted to evaluate the real-world applicability and impact of this simplified risk stratification approach.
