# Correction: Evaluating Blood Culture Parameters to Identify Patients at Low Risk of Infective Endocarditis Among Those With Bacteremia by Gram-positive Cocci

**DOI:** 10.1093/ofid/ofag618

**Published:** 2026-10-22

This is a correction to: Nicolas Fourré, Virgile Zimmermann, Nicoleta Ianculescu, Thomas Brahier, Zélie Dennebouy, André Teixeira-Antunes, Pierre Monney, Georgios Tzimas, Laurence Senn, Lars Niclauss, Matthias Kirsch, Benoit Guery, Matthaios Papadimitriou-Olivgeris, Evaluating Blood Culture Parameters to Identify Patients at Low Risk of Infective Endocarditis Among Those With Bacteremia by Gram-positive Cocci, *Open Forum Infectious Diseases*, Volume 12, Issue 9, September 2025, ofaf518, https://doi.org/10.1093/ofid/ofaf518

An error was identified in the population data in Table 3, where the original (incorrect) version listed the low-risk episodes as "398" instead of the corrected "382". Appropriate changes in Table 3 have been made and in related text (last paragraph of the Results and in section Discussion).

The following changes have been made to the originally published paper.

Errored data in Table 3 is corrected to read:

**Table 3.** Comparison of episodes classified as low- or high-risk for infective endocarditis among 2165 episodes with bacteremia by either *S. aureus*, streptococci or *E. faecalis*

	Low-risk^a^ (n=382)	High-risk^a^ (n=1783)	*P-*value
Male sex, n (%)	261 (68)	1266 (71)	0.294
Age (years), median (IQR)	66 (55-77)	69 (56-78)	0.270
Cardiac predisposition			
Intravenous drug use, n (%)	17 (5)	115 (6)	0.157
Prior infective endocarditis, n (%)	7 (2)	72 (4)	0.035
Prosthetic valve; surgical or transcatheter replacement, n (%)	26 (7)	209 (12)	0.005
Cardiac implantable electronic devices, n (%)	23 (6)	190 (11)	0.004
Manifestations			
Fever, n (%)	310 (81)	1526 (86)	0.034
Embolic events, n (%)	17 (5)	394 (22)	<0.001
Immunological phenomena, n (%)	1 (0.3)	52 (3)	0.001
Hematogenous native bone and joint infections, n (%)	17 (5)	280 (16)	<0.001
Pathogens			
*S. aureus*, n (%)	175 (46)	990 (56)	0.001
Low-risk streptococci,^b^ n (%)	39 (10)	235 (13)	0.127
High-risk streptococci,^c^ n (%)	113 (30)	287 (16)	<0.001
*E. faecalis*, n (%)	55 (14)	271 (15)	0.753
Infective endocarditis, n (%)	12 (3)	549 (31)	<0.001

IQR: interquartile range

^a^High-risk was considered those that had at least 2 positive out of 4 initial blood culture bottles or persistent bacteremia for at least 48h

^b^S. pneumoniae, S. pyogenes, S. agalactiae, S. dysgalactiae, S. salivarius, S. anginosus, S. constellatus and S. thermophilus

^c^all other streptococci not classified as low-risk

**Table 3.** Comparison of Episodes Classified as Low- or High-risk for Infective Endocarditis Among 2165 Episodes With Bacteremia by Either *S. Aureus*, Streptococci or *E. faecalis*

	Low-risk^a^ (n = 398)	High-risk^a^ (n = 1767)	*P V*alue
Male sex, n (%)	273 (69)	1254 (71)	.361
Age (y), median (IQR)	66 (55–77)	69 (56–78)	.270
Cardiac predisposition			
Intravenous drug use, n (%)	18 (5)	114 (7)	.164
Prior infective endocarditis, n (%)	7 (2)	72 (4)	.026
Prosthetic valve; surgical or transcatheter replacement, n (%)	26 (7)	209 (12)	.002
Cardiac implantable electronic devices, n (%)	24 (6)	189 (11)	.004
Manifestations			
Fever, n (%)	322 (81)	1514 (86)	.020
Embolic events, n (%)	24 (6)	387 (22)	<.001
Immunological phenomena, n (%)	1 (0.3)	52 (3)	<.001
Hematogenous native bone and joint infections, n (%)	17 (5)	280 (16)	<.001
Pathogens			
*S. aureus*, n (%)	185 (47)	980 (56)	.001
Low-risk streptococci,^b^ n (%)	41 (10)	233 (13)	.133
High-risk streptococci,^c^ n (%)	113 (28)	287 (16)	<.001
*E. faecalis*, n (%)	59 (15)	267 (15)	.938
Infective endocarditis, n (%)	14 (4)	547 (31)	<.001

Abbreviation: IQR, interquartile range.

^a^High-risk was considered those that had at least 2 positive out of 4 initial blood culture bottles or persistent bacteremia for at least 48 h.

^b^ *S. pneumoniae*, *S. pyogenes*, *S. agalactiae*, *S. dysgalactiae*, *S. salivarius*, *S. anginosus*, *S. constellatus*, and *S. thermophilus*.

^c^All other streptococci not classified as low-risk.

The final paragraph of section **Results**, is corrected to read: “Among the 382 episodes classified as low-risk for IE based the combination of only 1 positive out of 4 blood culture bottles and bacteremia clearance before 48 hours (Table 3), 26 (7%) had a prosthetic valve, 23 (6%) had a CIED, and 17 (5%) experienced embolic events. Twelve episodes (3%) among these low-risk cases were ultimately diagnosed with IE.” Instead: “Among the 398 episodes classified as low-risk for IE based the combination of only 1 positive out of 4 blood culture bottles and bacteremia clearance before 48 hours (Table 3), 26 (7%) had a prosthetic valve, 24 (6%) had a CIED, and 24 (6%) experienced embolic events. Fourteen episodes (4%) among these low-risk cases were ultimately diagnosed with IE.”

In section **Discussion**, 4th paragraph, 4th sentence, text is emended to read: “[…]6% had CIEDs, and 5% had embolic events,[…]” instead of: “[…]6% had CIEDs, and 6% had embolic events,[…]”.

instead of:

